# The effect of presymptomatic hypertension in posterior reversible encephalopathy syndrome

**DOI:** 10.1002/brb3.1190

**Published:** 2018-12-26

**Authors:** Moon Kyu Lee, Yang‐Je Cho, Seung‐Koo Lee, Sang Ku Jung, Kyoung Heo

In Lee, Cho, Lee, Jung, and Heo (2018), the affiliation of Moon Kyu Lee was published with incomplete details; it should have been “Department of Neurology, Gangneung Asan Hospital, Ulsan University College of Medicine, Gangneung, Korea.”
